# Impact of denosumab therapy on periapical healing following root canal treatment: a 10-year retrospective case-control study

**DOI:** 10.1186/s12903-026-09287-2

**Published:** 2026-08-03

**Authors:** Bokyung Shin, Ji-Young Yoon, Yeon-Jee Yoo, Won-Jun Shon

**Affiliations:** 1 Department of Conservative Dentistry, Section of Dentistry, Seoul National University Bundang Hospital, Seongnam, Republic of Korea; 2 Department of Conservative Dentistry, Dental Research Institute, Seoul National University School of Dentistry, Daehak-ro 101, Jongro-gu, Seoul, Republic of Korea

**Keywords:** Denosumab, Medication-related osteonecrosis of the jaw (MRONJ), Osteoporosis, Periapical index (PAI), Root canal treatment, Treatment outcome

## Abstract

**Introduction:**

Denosumab is a potent RANKL inhibitor used for osteoporosis, yet its impact on root canal treatment (RCT) outcomes remains less understood compared to bisphosphonates. This study evaluated the periapical healing outcomes of RCT in patients receiving denosumab and identified clinical variables influencing treatment success.

**Methods:**

This retrospective case-control study included 20 patients with osteoporosis receiving denosumab who underwent RCT at a major tertiary hospital between July 2014 and June 2024 (group D), along with 20 age- and sex-matched systemically healthy controls (group C). Among 116 denosumab-treated patients screened, 20 met the strict selection criteria and were enrolled as the study group. Periapical healing was assessed using the Periapical Index (PAI) at a minimum follow-up of 3 months after treatment. Various clinical and radiographic factors, including initial PAI scores and sealer extrusion, were analyzed using logistic regression to determine their impact on healing rates.

**Results:**

The median follow-up period was 534 (287–956) days for group D and 421 (235–1045) days for group C. The overall periapical healing rates were 75.0.% in the denosumab group and 90.0% in the healthy control group (*P* = 0.407). Multivariable logistic regression analysis did not identify any statistically significant predictors of healing outcomes. However, higher initial PAI scores (OR = 7.610, *P* = 0.088) and sealer extrusion (OR = 3.70, *P* = 0.185) were associated with less favorable outcomes in the multivariable model. Sealer extrusion was significantly associated with healing outcomes in univariate analysis (*P* = 0.034).

**Conclusions:**

Within the limitations of this study, there was no statistically significant difference in the proportion of healed cases between patients receiving denosumab therapy and healthy controls. These findings suggest that non-surgical root canal treatment may remain a viable tooth-preserving treatment option for patients receiving denosumab therapy. Careful apical control and long-term follow-up may be particularly important in patients presenting with large pre-existing periapical lesions and sealer extrusion.

**Supplementary Information:**

The online version contains supplementary material available at https://doi.org/10.1186/s12903-026-09287-2.

## Introduction

Root canal treatment (RCT) is a fundamental procedure aimed at removing and disinfecting infected pulp tissue to resolve pathological conditions of the pulp and periapical tissues. By preserving the natural dentition, RCT plays a crucial role in maintaining masticatory function and improving the overall quality of life for patients [1]. When primary RCT fails, nonsurgical retreatment or surgical intervention is attempted; however, if these measures prove unsuccessful, tooth extraction becomes the final, inevitable alternative [2].

The clinical success of RCT is primarily evaluated by the resolution of clinical symptoms and the radiographic healing of periapical lesions, often assessed using the Periapical Index (PAI) [3]. Since periapical healing is inherently a process of bone tissue regeneration, patients with systemic conditions affecting bone metabolism, such as osteoporosis (OP), may present unique challenges in post-treatment assessment [4, 5]. Despite the high global prevalence of OP, estimated at 18.3% [6], clinical studies specifically analyzing periapical healing in this population remain insufficient.

When RCT is unsuccessful, tooth extraction in osteoporotic patients carries a significant risk of Medication-related Osteonecrosis of the Jaw (MRONJ). According to the 2022 AAOMS position paper, the incidence of MRONJ following extraction in high-risk patients is reported to be approximately 1–5% [7]. To minimize the risk of MRONJ, contemporary clinical guidelines advocate for the preservation of teeth through RCT whenever possible [8, 9]. Therefore, achieving successful RCT outcomes and accurately evaluating healing dynamics in these patients is a critical clinical priority.

Recent literature has begun to explore the relationship between OP and periapical status. Cadoni et al. (2022) reported a higher incidence of periapical lesions in osteoporotic patients compared to healthy controls [5]; however, their study did not differentiate outcomes based on specific medication types, such as bisphosphonates (BPs) and denosumab. While a prospective study by Zamparini et al. (2021) found that BPs did not significantly impair RCT outcomes, it identified variables such as smoking and initial lesion size (PAI ≥ 3) as significant predictors of success [10].

To date, clinical evidence specifically evaluating the long-term success rates and prognostic factors of RCT in denosumab-treated patients remains extremely scarce. To the best of our knowledge, this study is among the first clinical investigations to longitudinally evaluate periapical healing outcomes and associated prognostic factors following root canal treatment in denosumab-treated patients. Therefore, the primary aim of this retrospective study was to evaluate the long-term outcomes of RCT in osteoporotic patients receiving denosumab at a major tertiary referral hospital over a 10-year period (2014–2024). Additionally, we sought to identify various clinical and radiographic prognostic factors—including pre-operative periapical status and the technical procedure variables—that may influence periapical healing in this population. Furthermore, this study compared these outcomes with a healthy control group to establish a more predictable prognosis for patients on antiresorptive therapy. The null hypothesis was that there would be no significant difference in periapical healing outcomes between the denosumab-treated patients and healthy controls.

## Materials and methods

### Study design and setting

This retrospective case-control study with age- and sex-matched controls was conducted to evaluate the impact of denosumab therapy on periapical healing outcomes following RCT in patients with osteoporosis. The “case” group was defined as osteoporotic patients under active denosumab therapy who underwent RCT for teeth with periapical lesions. To isolate the specific impact of denosumab on bone remodeling, the “control” group was established using systemically healthy individuals without osteoporosis or any history of bone-metabolism-modulating medications, tightly matched for age and sex.

Data were retrieved from the Clinical Data Warehouse (CDW) of Seoul National University Bundang Hospital (SNUBH) for the period between July 2014 and June 2024. The study protocol was approved by the Institutional Review Board (IRB) of SNUBH (IRB No. B-2408-919-102) and followed the principles of the Declaration of Helsinki (1964) and its subsequent amendments. The study adhered to the STROBE (Strengthening the Reporting of Observational Studies in Epidemiology) statement and checklist.

### Study size and case selection

Eligible participants were patients aged over 40-year with a confirmed diagnosis of primary OP who had received denosumab therapy. OP diagnosis was based on bone mineral density (BMD) measurement using dual-energy X-ray absorptiometry (DXA). Patients with diabetes, cardiovascular disease, autoimmune disease, cancer, osteoarticular conditions other than OP, or incomplete clinical documentation, were excluded.

The initial pool consisted of 116 patients extracted from the hospital’s Clinical Data Warehouse (CDW). After applying inclusion and exclusion criteria, 20 patients were included in the final analysis. Specific reasons for exclusion were as follows: RCT performed more than 6-month after denosumab injection (*n* = 53), no periapical dental radiograph follow-up (*n* = 24), RCT performed before denosumab injection (*n* = 13), presence of systemic comorbidities (*n* = 2), and a follow-up period of less than 3-month (*n* = 4). Thus, a total of 20 denosumab-treated patients with 20 treated teeth were enrolled in the study group (D group).

The D group was further divided into subgroups according to medication history:

- Denosumab only (D).
- Denosumab with prior bisphosphonate therapy (D + BP).
- Denosumab with prior selective estrogen receptor modulator therapy (D + SERM).

The control group was selected based on age (± 5 years) and sex distribution to resemble the D group. The control group was constructed through 1:1 frequency matching based on age (± 5 years) and sex to ensure comparability with the D group. To minimize selection bias, systemically healthy control subjects were randomly selected from the institutional database among patients who met the inclusion criteria within the same study period. A total of 20 patients (4 male, 16 female) with 20 teeth, with no history of osteoporosis, metabolic bone diseases, or any bone-modulating medications, were included in the control group (C group).

### Data collection

Patients’ medical and dental records were reviewed for demographic information, systemic health status, osteoporosis diagnosis and medications, clinical symptoms, and details of non-surgical root canal treatments. Additionally, functional factors that could influence healing outcomes, such as the presence of opposing dentition and the type of final restoration (e.g., direct resin or indirect crown), were recorded.

### Radiographic assessment

All available radiographs were reviewed. The PAI score was assessed by two trained and calibrated endodontists blinded to the group assignment. The PAI scores were independently assessed by both examiners, and any disagreements between the two examiners were resolved through discussion until a consensus was reached to ensure high inter-examiner reliability. For multi-rooted teeth, the highest PAI score among the multiple roots was designated as the representative score for the entire tooth after examiner consensus has been achieved.

The same examiners also assessed the quality of non-surgical endodontic treatment using the following criteria:

- Filling length: within 2 mm of the radiographic apex, underfilled, or overfilled.
- Sealer extrusion: presence or absence of apical extrusion.

Teeth were classified as either healed (PAI ≤ 2 and absence of clinical signs/symptoms) or diseased (PAI ≥ 3 and/or the presence of clinical signs or symptoms).

### Statistical analysis

Data was entered and managed using Microsoft Excel. Statistical analyses were conducted using SPSS version 27 (IBM Corp., Armonk, NY). Categorical variables were summarized as frequencies and percentages, and continuous variables as means ± standard deviations, medians, and ranges.

Group comparisons (D vs. C; D subgroups) were performed using Fisher’s exact test or Pearson’s chi-square test for categorical variables, and Mann-Whitney U test for continuous variables with non-normal distributions. Specifically, Fisher’s exact test was used compare the baseline prevalence of radiographic periapical lesions (pre-operative PAI ≥ 3) between the denosumab and control groups.

Additionally, comparability between the groups regarding functional factors—specifically the presence of opposing teeth and the type of final restoration—was verified using Fisher’s exact test.

A multivariable logistic regression model was used to identify factors associated with healed outcomes. Variables with *P* < 0.1 in univariate analysis (e.g., surgical history, pre-operative PAI, apical extrusion of sealer) were entered into the model. Adjusted odds ratios (ORs) and 95% confidence intervals were calculated using the Wald chi-square statistic.

Statistical significance was set at *P* < 0.05.

## Results

### Participants and baseline characteristics

A total of 40 patients were included in the final analysis: 20 in the Denosumab (D) group and 20 in the control (C) group (Table 1). Both groups were identical in sex distribution, consisting of 4 men (20.0%) and 16 women (80.0%) each. The mean age was 73.2 ± 8.3 years in the D group and 72.7 ± 8.3 years in the C group. There were no significant differences between the two groups in terms of tooth location, tooth type, or pre-operative clinical symptoms (all *P* > 0.05). The presence of opposing teeth showed no significant difference (*P* = 1.000), with only one tooth in the denosumab group lacking an opposing member. Similarly, the type of final restoration (direct vs. indirect) was statistically comparable between groups (*P* = 0.695).

**Table 1:** Baseline characteristics (*n* = 40)

Group	Denosumab (D)	Control (C)	*P*-value
Sex, number (%)			1.000
Male	4 (20.0)	4 (20.0)	
Female	16 (80.0)	16 (80.0)	
Age, mean ± SD	73.2 ± 8.3	72.7 ± 8.3	0.869
Maxilla-Mandible location (%)			0.343
Maxilla	8 (40.0)	12 (60.0)	
Mandible	12 (60.0)	8 (40.0)	
Tooth type (%)			0.421
Anterior	4 (20.0)	7 (35.0)	
Premolar	4 (20.0)	5 (25.0)	
Molar	12 (60.0)	8 (40.0)	
Opposing dentition (%)			1.000
Yes	19 (95.0)	20 (100.0)	
No	1 (5.0)	0 (0.0)	
Restoration type (%)			0.695
Indirect	15 (75.0)	17 (85.0)	
Direct	5 (25.0)	3 (15.0)	
Pre-op clinical symptoms (%)			0.176
Yes	16 (80.0)	11 (55.0)	
No	4 (20.0)	9 (45.0)	
Pre-op PAI (%)			0.977
1	9 (45.0)	7 (35.0)	
2	2 (10.0)	5 (25.0)	
3	4 (20.0)	4 (20.0)	
4	4 (20.0)	4 (20.0)	
5	1 (5.0)	0 (0)	
Pre-operative periapical lesion (PAI ≥ 3)	9 (45.0)	8 (40.0)	1.000
Endodontic treatment (%)			1.000
RCT	19 (95.0)	18 (90.0)	
Re-RCT	1 (5.0)	2 (10.0)	
Root canal filling quality (%)			1.000
Within 2 mm from apex	19 (95.0)	19 (95.0)	
Too short	1 (5.0)	1 (5.0)	
Over filling	0 (0)	0 (0)	
Apical extrusion of sealer (%)			1.000
Yes	5 (25.0)	4 (20.0)	
No	15 (75.0)	16 (80.0)	
Follow-up days, mean ± SD	758 ± 551 days	601 ± 417 days	0.330
Healed (%)	15 (75.0)	18 (90.0)	0.407

*Abbreviations*: *PAI* Periapical Index score, *Pre-op* Pre-operation, *RCT* Root Canal Treatment, *Re-RCT* Root Canal Retreatment, SD Standard Deviation

Data are presented as mean ± SD or number (%)

Regarding the baseline endodontic status, the prevalence of radiographically confirmed periapical lesions (defined as a pre-operative PAI score ≥ 3) was 45.0% (9/20 teeth) in the denosumab group and 40.0% (8/20 teeth) in the systemically healthy control group. This difference was not statistically significant (*P* = 1.000, Fisher’s exact test), indicating comparable baseline periapical conditions between the two groups prior to treatment. The D+SERM subgroup included only one patient and was therefore interpreted descriptively.

### Radiographic healing outcomes

**Fig. 1:**
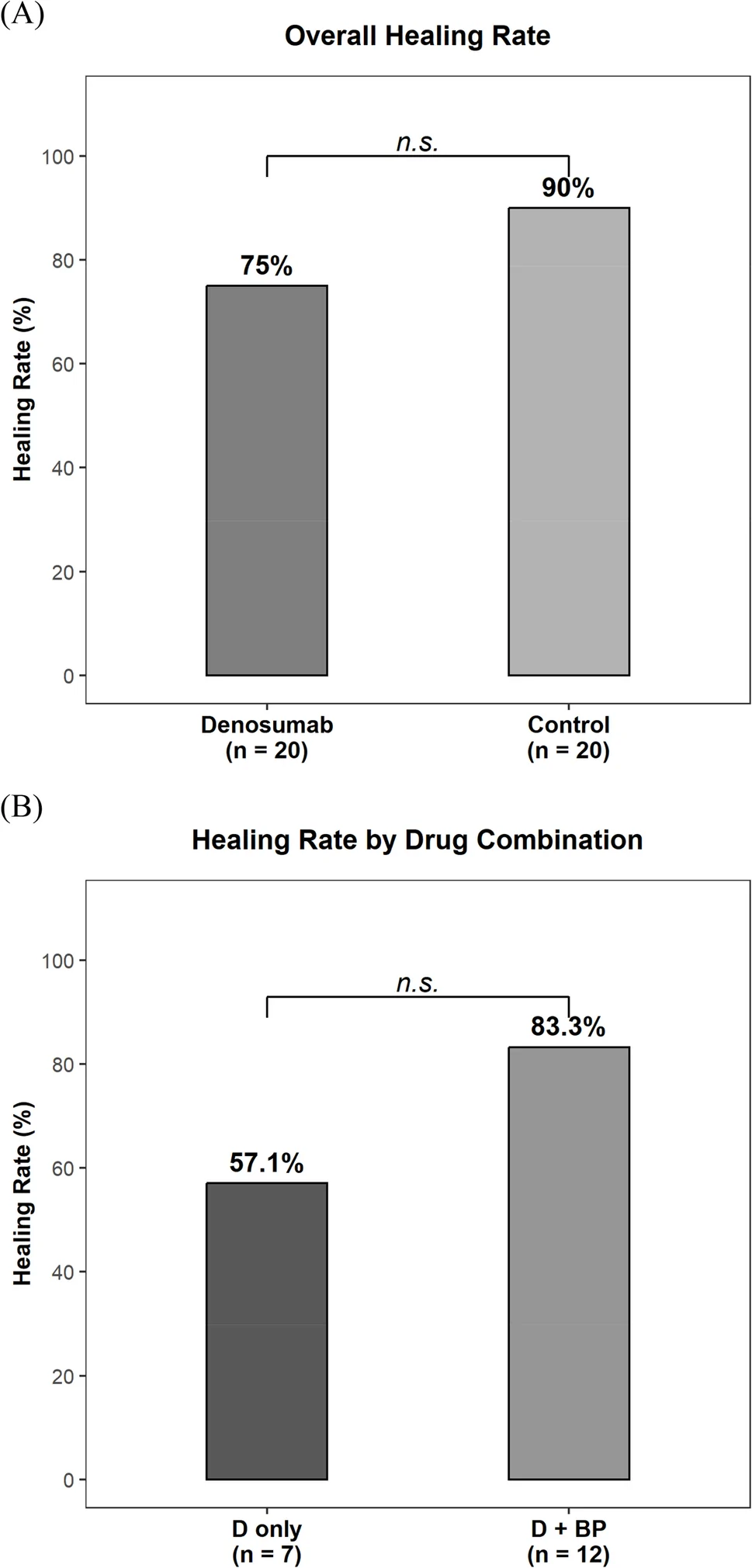
Comparison of periapical healing rates. **A** Overall healing rate: comparison between the denosumab group (*n* = 20) and the healthy control group (*n* = 20). The healing rates were 75.0% in the denosumab group and 90.0% in the control group, with no statistically significant difference between groups (*P* = 0.407 by Fisher’s exact test). **B** Healing Rate by Medication History: Subgroup analysis within the denosumab group based on prior medication history. The healing rates were 57.1% in the denosumab-naive group (D-only, *n* = 7) and 83.3% in the medication-transition group (D + BP, *n* = 12); however, the difference was not statistically significant (*P* = 0.479). The D+SERM subgroup included only one patient and is presented for descriptive completeness only. Abbreviations: n.s., not statistically significant (P > 0.05)

The overall healing rate was 75% in the D group and 90% in the C group (*P* = 0.407) (Fig. 1A). Within the D group, the healing rate was 90.9% (10/11) for teeth with a pre-operative PAI score of ≤ 2, and 55.6% (5/9) for those with a score of ≥ 3 (*P* = 0.127).

**Fig. 2:**
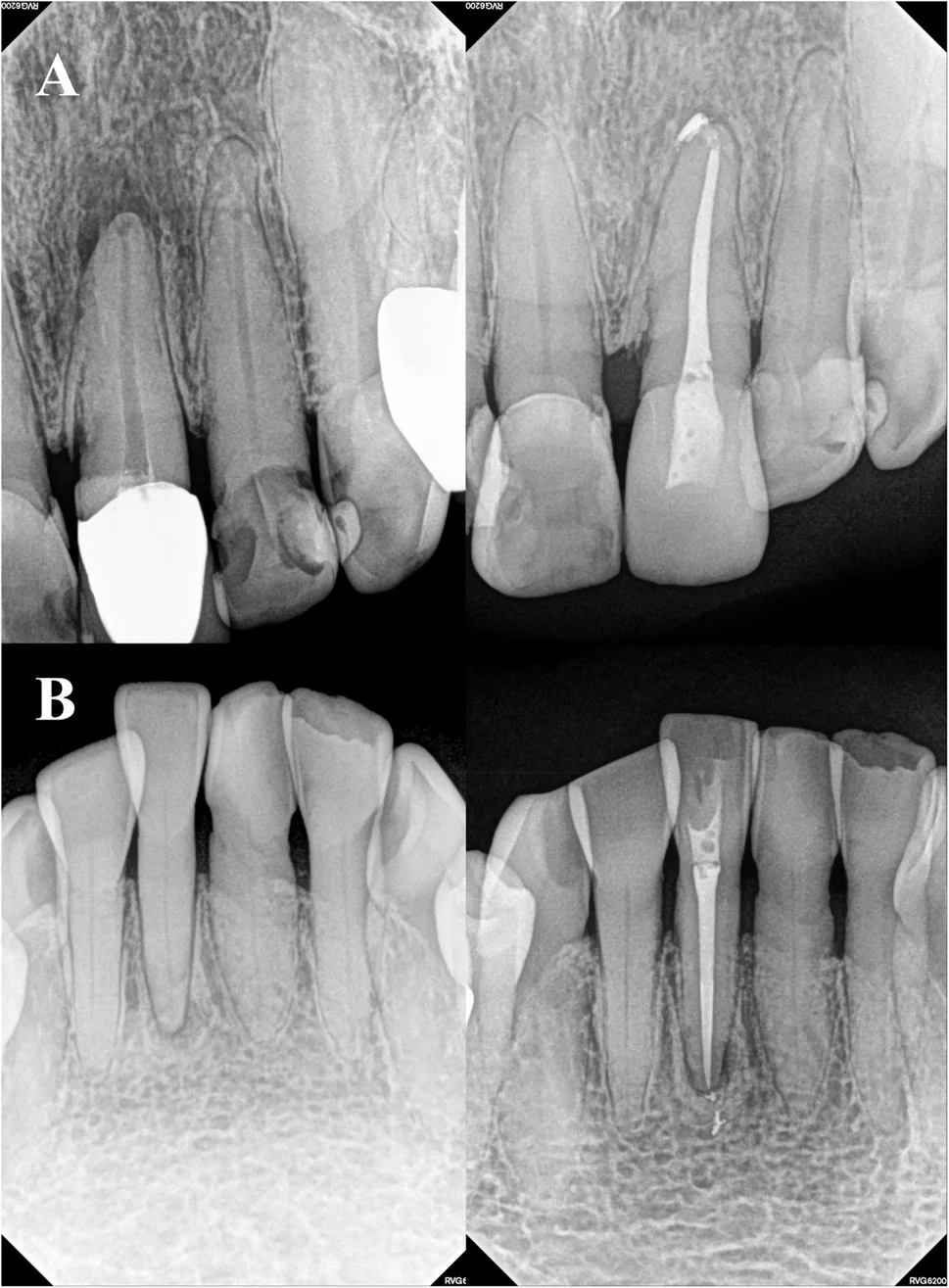
Representative healed and diseased cases demonstrated by periapical radiographs. **A** Healed case: initial pre-operative Periapical Index (PAI) score of 5 (left) decreased to a final PAI score of 2 (right) after treatment, indicating substantial resolution of the periapical lesion, bone regeneration, and a favorable overall treatment outcome. **B** Diseased case: initial pre-operative PAI score of 3 (left) remained unchanged at a final PAI score of 3 (right) after treatment, demonstrating persistent periapical radiolucency at follow-up and an unfavorable overall treatment outcome

Representative radiographs illustrating the radiographic outcome assessment are presented in (Fig. 2). Figure 2A demonstrates a representative healed case showing substantial resolution of a periapical lesion, whereas Fig. 2B demonstrates a representative diseased case with persistent periapical radiolucency at follow-up.

All five failure cases in the denosumab group occurred in female patients.

### Univariate analysis of healing outcomes

The associations between clinical variables and healing status are presented in (Table 2). In the univariate analysis, the proportion of healed cases was 85.7% (30/35) in patients without a history of surgery and 60.0% (3/5) in those with a surgical history, although the difference was not statistically significant (*P* = 0.204). A significant association was observed between pre-operative PAI scores and healing outcome (95.7% for PAI ≤ 2 vs. 64.7% for PAI ≥ 3; *P* = 0.030). Apical extrusion of sealer was also associated with healing status (*P* = 0.034). The healed rate was 90.3% (28/31) in patients without extrusion compared to 55.6% (5/9) in those with extrusion. Other clinical variables, including duration of osteoporosis, and follow-up period, were not significantly associated with the healing outcome (all *P* > 0.05).

**Table 2:** Univariate analysis of clinical variables associated with healed outcome (*n* = 40)

Variables	Healed = Yes (*n*, %)	Healed = No (*n*, %)	*P* value
Demographic Factors			
Sex (Male/Female)	8 (24.2%) / 25(75.8%)	0 (0.0%) / 7 (100.0%)	0.309
Age (years, Median [IQR])	76 [67–79]	77.0 [70.5–78.5]	0.654^‡^
Tooth-related Factors			
Location (Maxilla/Mandible)	17 (51.5%) / 16 (48.5%)	3 (42.9%) / 4 (57.1%)	1.000
Tooth type (Ant./Pre./Molar)	8 / 7 / 18	3 / 2 / 2	0.439
Clinical & Procedural Factors			
Pre-op symptoms (yes/no)	23 (69.7%) / 10 (30.3%)	4 (57.1%) / 3 (42.9%)	0.662
Pre-op PAI			0.030^*^
Low (≤ 2)	22 (66.7)	1 (14.3)	
High (≥ 3)	11 (33.3)	6 (85.7)	
Apical extrusion of sealer			0.034^*^
Yes	5 (15.2)	4 (57.1)	
No	28 (84.8)	3 (42.9)	
Quality of filling			0.447
1. Within 2 mm from apex	31 (93.9)	7 (100.0)	
2. Too short	2 (6.1)	0 (0.0)	
3. Overfilling	0 (0.0)	0 (0.0)	
Surgical history (no/yes)	30 (90.9%) / 3 (9.1%)	5 (71.4%) / 2 (28.6%)	0.204
Duration of disease (days, Median [IQR])	2126 [601–3876]	602 [349–1422]	0.493^§^
Med-to-treatment interval (days, Median [IQR]) ^†^	106 [46.5–137]	112 [41–126]	1.000^§^
Follow-up period (days, Median [IQR])	534 [287–956]	421 [235–1045]	0.887^‡^

*Abbreviations*: *Ant* Anterior, *IQR* Interquartile Range, *PAI* Periapical Index score, *Pre* Premolar, *Pre-op* Pre-operation

* *P* < 0.05 by Fisher’s Exact Test. **†** interval between the last medication and the start of root canal treatment, analyzed within the denosumab group (*n* = 20). ‡ Analyzed by Mann-Whitney U test. § Analyzed within the denosumab group (*n* = 20) using Mann-Whitney U test

### Multivariate analysis of healing outcomes

Multivariable logistic regression was conducted to assess the influence of clinical factors on healing (Table 3). The model included surgical history, apical extrusion of sealer, and pre-operative PAI (dichotomized as ≤ 2 vs. ≥ 3).

**Table 3:** Multivariate logistic regression analysis of factors associated with healing (*n* = 40)

Pre-op PAI (≥ 3 vs. ≤ 2)	OR	*P* value	95% CI
	7.610	0.088	0.737—78.583
Apical extrusion of sealer (yes vs. no)	3.700	0.185	0.534—25.616
Surgical history (yes vs. no)	1.606	0.678	0.171—15.038

*Abbreviations*: *CI* Confidence Interval, *OR* Odds Ratio, *PAI* Periapical Index score, *Pre-op* Pre-operation

Hosmer-Lemeshow Test: *P* > 0.05 (Model fit is appropriate). χ² (3) = 8.957, *P* = 0.030, Nagelkerke *R²* = 0.332. Overall prediction accuracy = 87.5%. *P* < 0.1 indicates marginal significance

The regression model was statistically significant (χ² (3) = 8.957, *P* = 0.030). The explained variance was moderate (Nagelkerke *R²* = 0.332). The overall prediction accuracy of the model was 87.5%.

**Fig. 3:**
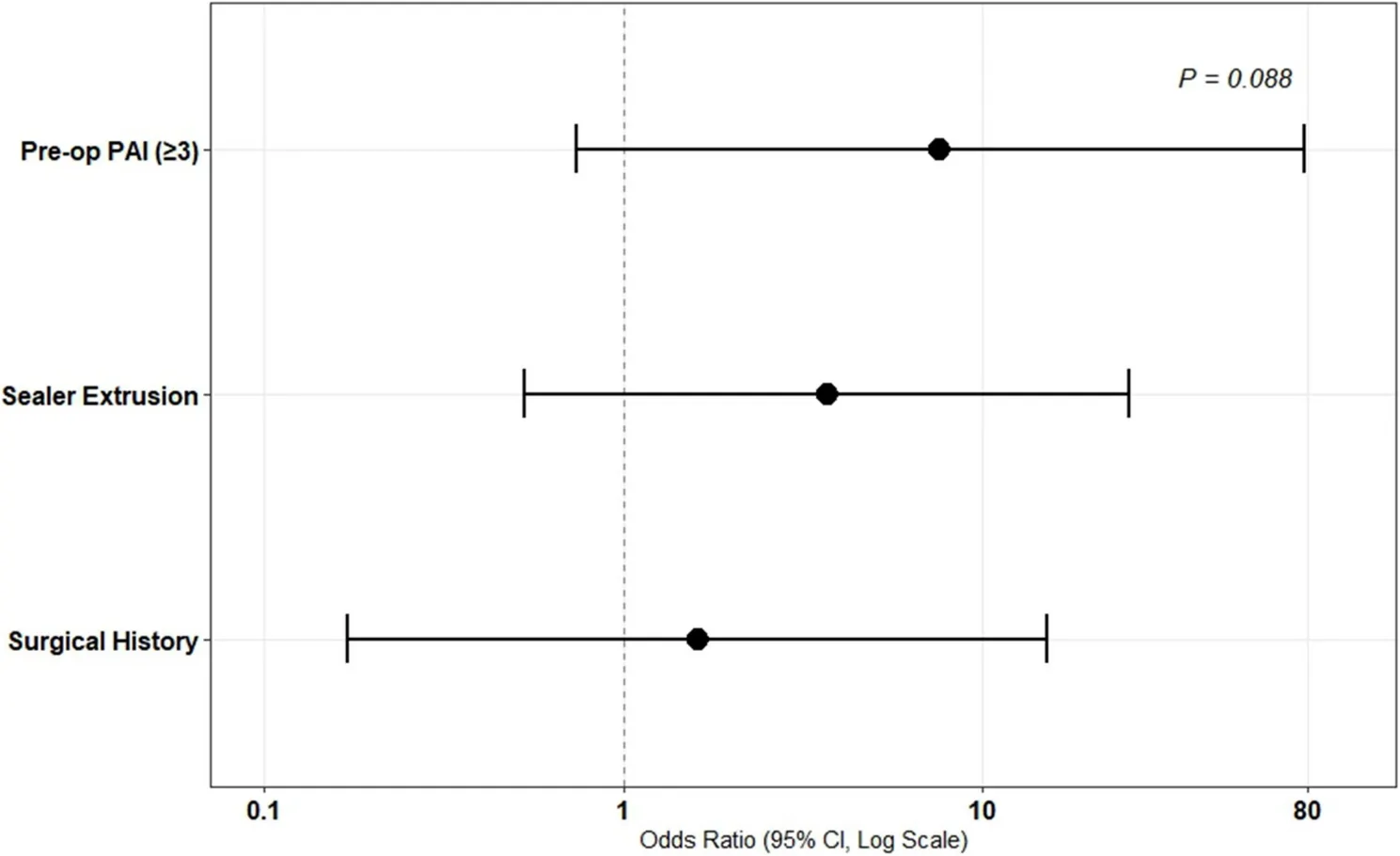
Forest plot of prognostic factors for periapical healing. The plot illustrates the odds ratios (ORs) and 95% confidence intervals (CIs) derived from the multivariable logistic regression. Clinical variables, including pre-operative PAI score, tooth location, and presence of sealer extrusion were analyzed. A high pre-operative PAI score (≥ 3) showed an OR of 7.610 (*P* = 0.088). Abbreviations: CI, confidence interval; OR, odds ratio; PAI, Periapical Index.

In the multivariable model, a high pre-operative PAI score (≥ 3) was associated with less favorable healing outcomes, although the association did not achieve statistical significance (OR = 7.610, 95% CI: 0.737—78.583, *P* = 0.088) (Fig. 3). Additionally, apical extrusion of sealer was associated with less favorable healing outcomes (OR = 3.70, 95% CI: 0.534—25.616, *P* = 0.185). No individual predictor reached statistical significance in the multivariable model.

### Subgroup analysis according to medication history

Subgroup analysis within the denosumab group (*n* = 20) was performed according to medication history. Radiographic healing was observed in 4 out of 7 patients (57.1%) in the D-only subgroup and 10 out of 12 patients (83.3%) in the D + BP subgroup. The D+SERM subgroup included only a single patient who exhibited successful healing. No statistically significant difference in healing outcomes was found among these medication subgroups. (*P* = 0.479, Fisher-Freeman-Halton exact test).

## 4. Discussion

This retrospective case-control study was conducted to evaluate the periapical healing outcomes following RCT in patients with osteoporosis undergoing denosumab therapy compared to healthy controls. Although a lower healing rate was observed in the denosumab group (75.0%) compared to the control group (90.0%), this difference did not reach statistical significance (*P* = 0.407). Accordingly, the null hypothesis—that there would be no significant difference in periapical healing outcomes between the denosumab group and the control group—was accepted. Within the limitations of this study, the findings suggest that initial periapical status and the clinical quality of the endodontic procedure may be associated with healing outcomes. Specifically, univariate analysis revealed that higher pre-operative PAI scores (*P* = 0.030) and the presence of apical sealer extrusion (*P* = 0.034) were significantly associated with lower rates of healing. Furthermore, in the multivariable logistic regression model, a high pre-operative PAI score (PAI ≥ 3) was associated with less favorable healing, although it did not reach statistical significance (OR = 7.610, *P* = 0.088). Therefore, these findings should be interpreted as exploratory and suggest that conventional prognostic factors, such as initial PAI and procedural quality, may remain clinically relevant in patients receiving denosumab.

OP is a prevalent skeletal disease, with a global prevalence recently estimated at 18.3% according to a comprehensive systematic review and meta-analysis [6]. Characterized by the disruption of bone microarchitecture, OP affects the whole body, including the jaws, and is frequently associated with low estrogen levels [11]. Interestingly, in our study, all 5 failure cases in the denosumab group occurred in female patients. Given the small number of failure cases, this observation should be interpreted cautiously. However, the predominance of postmenopausal women in the denosumab-treated population may partly explain this finding. Patients with OP exhibit an increased risk of periodontitis and accelerated tissue destruction [4, 12]. This systemic condition has a destructive effect on bone tissue through multiple mechanisms, including not only the RANKL pathway but also the NLRP3/Caspase-1/IL-1β cascade, which contributes to faster bone resorption and a more rapid progression of apical periodontitis compared to healthy individuals [11]. In addition, although the lower age inclusion threshold was set at > 40 years in accordance with previous osteoporosis-related studies, the final cohort demonstrated a mean age above 70 years, reflecting the predominantly elderly population receiving denosumab therapy in real-world clinical practice.

Interestingly, our baseline data showed that the prevalence of radiographically detectable periapical lesions (PAI ≥ 3) was similar between patients receiving denosumab therapy and healthy controls (45.0% vs. 40.0%, *P* = 1.000, Fisher’s exact test). Within the limitations of this study, no statistically significant difference in baseline radiographic periapical status was observed between the two groups. Previous experimental studies have suggested that RANKL inhibition suppresses osteoclast-mediated bone resorption during the early stages of pulpal infection, potentially influencing the radiographic manifestation of periapical lesions [13–15]. However, the clinical implications of these experimental findings remain unclear, and further studies are needed to clarify the influence of denosumab on both the development and healing of periapical lesions.

The biological mechanism underlying the delayed or impaired healing in denosumab-treated patients may be attributed to its function as a high-affinity and highly specific monoclonal antibody for RANKL [16]. By mimicking the natural decoy receptor osteoprotegerin (OPG), denosumab interferes with the RANK-RANKL-OPG pathway, a key regulator of bone metabolism [17], thereby preventing the maturation and activation of osteoclasts [18].

Unlike bisphosphonates, which are incorporated into the bone matrix, denosumab provides a potent but reversible anti-resorptive effect; however, this continuous suppression of bone turnover significantly may theoretically limit the bone remodeling required for the resolution of periapical lesions [16].

Since periapical healing is a dynamic process necessitating the removal of necrotic and inflammatory tissues—a phase that involves high RANKL expression related to the severity of apical periodontitis [13, 14, 19]—the potent antiresorptive action of denosumab is suggested to potentially suppresses the necessary remodeling cycle [16, 20, 21].

Our findings may be interpreted in the context of previous experimental evidence from animal models. Aghaloo et al. [15] and Song et al. [22] demonstrated that RANKL inhibition induces jaw-like lesions specifically in the presence of periapical inflammation by preventing normal bone resorption and leading to disorganized bone formation. While a recent animal study by Lopes et al. [23] suggested that apical patency during antiresorptive therapy does not necessarily increase the risk of osteonecrosis, clinical radiographic evidence has consistently associated persistent periapical radiolucency and unsatisfactory endodontic treatment with a higher risk of MRONJ [24]. Notably, a recent scoping review by Mauceri et al. [25] confirms that failed RCT itself can serve as a trigger for MRONJ onset, especially when associated with technical errors like overfilling.

Consistent with previous studies on bisphosphonates (BPs) by Hsiao et al. [26] and Zamparini et al. [10], which reported that BP therapy does not significantly impair RCT success rates, our findings also showed no statistically significant difference in healing outcomes between denosumab-treated patients and healthy controls.

Although the healing rate was numerically lower in the denosumab group than in the control group (75.0% vs. 90.0%), this discrepancy was not statistically significant. The distinct mechanisms of action of denosumab and bisphosphonates may represent a potential biological explanation that warrants further investigation. Denosumab provides a more immediate and potent extracellular RANKL inhibition, whereas BPs bind to the bone matrix and primarily affect osteoclasts during the resorption phase [16, 21].

Our subgroup analysis showed a higher healed rate in the medication-transition group (D + BP group, 83.3%) compared to the denosumab-only (denosumab-naive) group (57.1%), although the difference was not statistically significant (*P* = 0.479). Given the limited sample size, these findings should be interpreted cautiously. Nevertheless, the distinct pharmacological mechanisms of denosumab and bisphosphonates may represent a potential biological explanation that warrants further investigation. Unlike BPs, which primarily inhibit osteoclasts during the bone resorption phase and bind avidly to the bone mineral with an effect lasting months to years, denosumab leads to a more direct extracellular inhibition of RANKL and reversible suppression of bone remodeling [16, 21, 27]. Whether these mechanistic differences influence periapical healing outcomes remains to be clarified in larger studies.

In contrast, the “residual effect” of prior BP therapy in the transition group may represent another potential explanation for the observed numerical difference in healing. Previous studies have suggested that the prior BP exposure may influence subsequent bone remodeling dynamics during antiresorptive therapy [5, 16, 27]. However, the present subgroup analysis was limited by the small sample size and heterogeneous medication histories. Therefore, these findings should be considered exploratory and interpreted with caution until validated in larger studies.

From a clinical perspective, RCT remains the preferred alternative to extraction to minimize MRONJ risk [28, 29]. This is supported by the American Association of Endodontists (AAE) Position Statement, which recommends RCT to avoid invasive surgery [30]. However, the AAE also cautions that procedures requiring “exposure and manipulation of the bone” could carry a risk of MRONJ comparable to extraction [30]. Therefore, before initiating dental treatment, it is essential for the practitioner to take a comprehensive medical history, specifying the etiology of OP, the bone mineral density of each patient, and the specific medications they are taking [11]. In the absence of specialized endodontic guidelines, clinicians must adhere to the following technical precautions [25, 28]:

1. Strict Apical Control: AAE guidelines emphasize avoiding instrumentation beyond the apex [30]. As identified in our study, patients with larger initial lesions (PAI ≥ 3) tended to show less favorable healing outcomes in the present study. This highlights the importance of achieving precise apical control to prevent mechanical irritation of the potentially suppressed periapical bone [25].
2. Prevention of Sealer Extrusion: Our univariate analysis identified an association between sealer extrusion and less favorable healing outcomes (*P* = 0.034), echoing expert opinions that overfilling should be avoided to minimize chronic inflammation [25]. In a pharmacologically suppressed environment, extruded materials may act as a source of persistent irritants, underscoring the importance of careful obturation techniques.

Several limitations should be carefully considered when interpreting our findings.

First, the primary limitation is the small sample size (*n* = 20 per group, total *n* = 40). Due to strict exclusion criteria during electronic chart screening—such as excluding 53 patients whose root canal treatments were performed more than 6 months after the last denosumab administration—the final eligible cohort was substantially reduced. Consequently, the statistical power of the analyses was limited, and the possibility of Type II errors cannot be excluded. This limitation is reflected in the wide confidence intervals (e.g., 95% CI: 0.737–78.583) and marginal P-values observed in the multivariable logistic regression. Although the odds ratio (7.610) may indicate a potential association between severe initial periapical status and less favorable healing outcomes, the extremely wide confidence interval indicates low statistical precision due to the small sample size; thus, this finding must be interpreted with appropriate caution. While a single-arm observational design could have been adopted to evaluate healing outcomes within the denosumab cohort alone, we included an age- and sex-matched healthy comparison group to provide a clinical reference for interpreting the observed healing outcomes.

Second, because the number of outcome events was limited, entering multiple predictors derived from a univariate screening threshold of *P* < 0.1 into the multivariable model carries an inherent risk of model overfitting. Furthermore, formal correction for multiple testing was not applied. Therefore, non-significant findings–such as the difference in healing rates between the denosumab and control groups (75.0% vs. 90.0%, *P* = 0.407) –should be interpreted as exploratory observations rather than definitive conclusions.

Third, the medication history subgroups within the denosumab cohort were extremely small (D-only: *n* = 7, D + BP: *n* = 12, D+SERM: *n* = 1), which limits the statistical generalizability of the subgroup analyses. While the numerical disparity in healing rates was observed (57.1% vs. 83.3%), the extremely small subgroup sizes preclude meaningful interpretation of this finding.

Fourth, although the study followed a strict inclusion criterion of at least 3 months of follow-up, the durations still varied among patients. Specifically, the median follow-up period was longer in the denosumab group than in the control group (534 days vs. 421 days). Because radiographic healing generally increases with longer observation periods, this difference in follow-up duration may have influenced the observed healing rates. Nevertheless, the non-standardized follow-up periods and treatment intervals remain important methodological limitations and inevitably restrict our ability to draw definitive causal inferences from the present findings.

In addition, because the highest PAI score among the roots was used to represent multi-rooted teeth, a slight tendency toward overestimation of periapical severity cannot be entirely excluded.

Finally, due to the retrospective nature of this electronic chart review, the potential for selection bias cannot be entirely eliminated despite age- and sex-matching. Furthermore, the potential influence of functional and clinical factors—such as occlusal loading, the type of final restoration, and mild or transient clinical symptoms that might have been underreported—could not be fully standardized, which might overestimate the successful healing rates.

Future prospective, large-scale multicenter studies with larger sample sizes, formal a priori power calculations, and standardized treatment protocols―including precise tracking of denosumab dosing details and administration schedules―are warranted to validate these preliminary observations and establish more robust evidence-based conclusions.

## Conclusion

Within the limitations of this study, there was no statistically significant difference in the proportion of healed cases between patients receiving denosumab therapy and healthy controls. These findings suggest that non-surgical RCT may remain a viable tooth-preserving treatment option for patients receiving denosumab therapy. Higher pre-operative PAI scores and apical sealer extrusion were associated with less favorable healing outcomes. Conventional endodontic prognostic factors, such as initial periapical status and procedural quality, may therefore remain clinically relevant in this patient population. Careful apical control and long-term follow-up may be particularly important when managing these patients. Further large-scale studies are necessary to validate these findings and better characterize endodontic outcomes in patients receiving denosumab therapy.

## Supplementary Information

Supplementary Material 1.

## Data Availability

The datasets generated and/or analyzed during the current study are not publicly available due to containing information that could compromise the privacy of research participants but are available from the corresponding author on reasonable request.

## Abbreviations

AAE: American Association of Endodontists
BMD: Bone mineral density
BP: Bisphosphonate
CDW: Clinical Data Warehouse
D: Denosumab
DXA: Dual-energy X-ray absorptiometry
IQR: Interquartile range
MRONJ: Medication-related osteonecrosis of the jaw
OP: Osteoporosis
OR: Odds ratio
PAI: Periapical index
RANKL: Receptor activator of nuclear factor kappa-B ligand
RCT: Root canal treatment

## References

[1] Gulabivala K, Ng YL. Factors that affect the outcomes of root canal treatment and retreatment-a reframing of the principles. Int Endod J. 2023;56(Suppl 2):82–115. 36710532 10.1111/iej.13897

[2] de Chevigny C, Dao TT, Basrani BR, Marquis V, Farzaneh M, Abitbol S, et al. Treatment outcome in endodontics: the Toronto study–phase 4: initial treatment. J Endod. 2008;34(3):258–63. 18291271 10.1016/j.joen.2007.10.017

[3] Orstavik D, Kerekes K, Eriksen HM. The periapical index: a scoring system for radiographic assessment of apical periodontitis. Endod Dent Traumatol. 1986;2(1):20–34. 3457698 10.1111/j.1600-9657.1986.tb00119.x

[4] Yu B, Wang CY. Osteoporosis and periodontal diseases - an update on their association and mechanistic links. Periodontol 2000. 2022;89(1):99–113. 35244945 10.1111/prd.12422 PMC9067601

[5] Cadoni E, Ideo F, Marongiu G, Mezzena S, Frigau L, Mela Q, et al. Periapical status in patients affected by osteoporosis: a retrospective clinical study. Clin Exp Dent Res. 2022;8(5):1068–75. 35698910 10.1002/cre2.604 PMC9562578

[6] Salari N, Ghasemi H, Mohammadi L, Behzadi MH, Rabieenia E, Shohaimi S, et al. The global prevalence of osteoporosis in the world: a comprehensive systematic review and meta-analysis. J Orthop Surg Res. 2021;16(1):609. 34657598 10.1186/s13018-021-02772-0 PMC8522202

[7] Ruggiero SL, Dodson TB, Aghaloo T, Carlson ER, Ward BB, Kademani D. American association of oral and maxillofacial surgeons’ position paper on medication-related osteonecrosis of the jaws-2022 Update. J Oral Maxillofac Surg. 2022;80(5):920–43. 35300956 10.1016/j.joms.2022.02.008

[8] Moinzadeh AT, Shemesh H, Neirynck NA, Aubert C, Wesselink PR. Bisphosphonates and their clinical implications in endodontic therapy. Int Endod J. 2013;46(5):391–8. 23137312 10.1111/iej.12018

[9] Kyrgidis A, Arora A, Lyroudia K, Antoniades K. Root canal therapy for the prevention of osteonecrosis of the jaws: an evidence-based clinical update. Aust Endod J. 2010;36(3):130–3. 21140589 10.1111/j.1747-4477.2010.00280.x

[10] Zamparini F, Pelliccioni GA, Spinelli A, Gissi DB, Gandolfi MG, Prati C. Root canal treatment of compromised teeth as alternative treatment for patients receiving bisphosphonates: 60-month results of a prospective clinical study. Int Endod J. 2021;54(2):156–71. 32901962 10.1111/iej.13405

[11] Stanev E, Vasileva RI. Influence of osteoporosis on the course of apical periodontitis. Eur J Dent. 2024;18(4):997–1003. 38759999 10.1055/s-0044-1785533 PMC11479746

[12] Tilotta F, Gosset M, Herrou J, Briot K, Roux C. Association between osteoporosis and periodontitis. Joint Bone Spine. 2025;92(4):105883. 40090617 10.1016/j.jbspin.2025.105883

[13] Belibasakis GN, Rechenberg DK, Zehnder M. The receptor activator of NF-κB ligand-osteoprotegerin system in pulpal and periapical disease. Int Endod J. 2013;46(2):99–111. 22900632 10.1111/j.1365-2591.2012.02105.x

[14] Wang Q, Wang L, Sheng L, Zhang B, Jieensi B, Zheng S, et al. Correlation between PD-1/PD-L1 and RANKL/OPG in chronic apical periodontitis model of Sprague-Dawley rats. Odontology. 2024;112(4):1113–22. 38528238 10.1007/s10266-024-00911-7

[15] Aghaloo TL, Cheong S, Bezouglaia O, Kostenuik P, Atti E, Dry SM, et al. RANKL inhibitors induce osteonecrosis of the jaw in mice with periapical disease. J Bone Min Res. 2014;29(4):843–54. 10.1002/jbmr.2097 PMC4476544 24115073

[16] Ferrari S, Langdahl B. Mechanisms underlying the long-term and withdrawal effects of denosumab therapy on bone. Nat Rev Rheumatol. 2023;19(5):307–17. 37024711 10.1038/s41584-023-00935-3

[17] Lacey DL, Boyle WJ, Simonet WS, Kostenuik PJ, Dougall WC, Sullivan JK, et al. Bench to bedside: elucidation of the OPG-RANK-RANKL pathway and the development of denosumab. Nat Rev Drug Discov. 2012;11(5):401–19. 22543469 10.1038/nrd3705

[18] Schieferdecker A, Voigt M, Riecken K, Braig F, Schinke T, Loges S, et al. Denosumab mimics the natural decoy receptor osteoprotegerin by interacting with its major binding site on RANKL. Oncotarget. 2014;5(16):6647–53. 25138051 10.18632/oncotarget.2160 PMC4196153

[19] Braz-Silva PH, Bergamini ML, Mardegan AP, De Rosa CS, Hasseus B, Jonasson P. Inflammatory profile of chronic apical periodontitis: a literature review. Acta Odontol Scand. 2019;77(3):173–80. 30585523 10.1080/00016357.2018.1521005

[20] McDonald MM, Khoo WH, Ng PY, Xiao Y, Zamerli J, Thatcher P, et al. Osteoclasts recycle via osteomorphs during RANKL-stimulated bone resorption. Cell. 2021;184(5):1330–e4713. 33636130 10.1016/j.cell.2021.02.002 PMC7938889

[21] Baron R, Ferrari S, Russell RG. Denosumab and bisphosphonates: different mechanisms of action and effects. Bone. 2011;48(4):677–92. 21145999 10.1016/j.bone.2010.11.020

[22] Song M, Alshaikh A, Kim T, Kim S, Dang M, Mehrazarin S, et al. Preexisting periapical inflammatory condition exacerbates tooth extraction-induced bisphosphonate-related osteonecrosis of the jaw lesions in mice. J Endod. 2016;42(11):1641–6. 27637460 10.1016/j.joen.2016.07.020 PMC5085836

[23] Lopes CF, Pinto KP, Ferreira CMA, de Souza JB, Lopes RT, Alves A, et al. The risk of osteonecrosis after apical patency during antiresorptive therapy in an animal model. Int Endod J. 2025;58(5):776–86. 39904856 10.1111/iej.14207

[24] Gaêta-Araujo H, Ferreira Leite A, de Faria Vasconcelos K, Coropciuc R, Politis C, Jacobs R, et al. Why do some extraction sites develop medication-related osteonecrosis of the jaw and others do not? a within-patient study assessing radiographic predictors. Int J Oral Implantol (Berl). 2021;14(1):87–98. 34006074

[25] Mauceri R, Coppini M, Caponio VCA, Zamparini F, Prati C, Campisi G. Endodontic therapy and Medication-related osteonecrosis of the jaw onset: a scoping review and expert opinion-based qualitative meta-synthesis. BMC Oral Health. 2025;25(1):1362. 40849463 10.1186/s12903-025-06741-5 PMC12374311

[26] Hsiao A, Glickman G, He J. A retrospective clinical and radiographic study on healing of periradicular lesions in patients taking oral bisphosphonates. J Endod. 2009;35(11):1525–8. 19840641 10.1016/j.joen.2009.07.020

[27] Reid IR, Billington EO. Drug therapy for osteoporosis in older adults. Lancet. 2022;399(10329):1080–92. 35279261 10.1016/S0140-6736(21)02646-5

[28] Song M. Dental care for patients taking antiresorptive drugs: a literature review. Restor Dent Endod. 2019;44(4):e42. 31799170 10.5395/rde.2019.44.e42 PMC6875544

[29] Boston B, Ipe D, Capitanescu B, Gresita A, Hamlet S, Love R, et al. Medication-related osteonecrosis of the jaw: a disease of significant importance for older patients. J Am Geriatr Soc. 2023;71(8):2640–52. 37224415 10.1111/jgs.18414

[30] American association of endodontists. AAE position statement: endodontic implications of medication-related osteonecrosis of the jaw. Chicago, IL: American association of endodontists; 2018.

