# Supplementary material for: Impact of denosumab therapy on periapical healing following root canal treatment: a 10-year retrospective case-control study
Source: BMC Oral Health. 2026 Aug 3;26:1708. doi: 10.1186/s12903-026-09287-2 (PMC13563897; doi:10.1186/s12903-026-09287-2)
Supplement: Supplementary file 1 [file 12903_2026_9287_MOESM1_ESM.docx]

**STROBE check list for case-control studies**

|  | **Item No** | **Recommendation** | **Reported**  **on Page** | | **Relevant text/Comments** |
| --- | --- | --- | --- | --- | --- |
| **Title and abstract** | 1 | (a) Indicate the study’s design with a commonly used term in the title or the abstract | Page 1-2 | |  |
|  |  | (b) Provide in the abstract an informative and balanced summary of what was done and what was found | Page 2 |  | |
| **Introduction** |  |  |  | |  |
| Background/rationale | 2 | Explain the scientific background and rationale for the investigation being reported | Page 4 | |  |
| Objectives | 3 | State specific objectives, including any prespecified hypotheses | Page 5 | |  |
| **Methods** |  |  |  | |  |
| Study design | 4 | Present key elements of study design early in the paper | Page 5 | |  |
| Setting | 5 | Describe the setting, locations, and relevant dates, including periods of recruitment, exposure, follow-up, and data collection | Page 5-6 | |  |
| Participants | 6 | (a) Give the eligibility criteria, and the sources and methods of case ascertainment and control selection. Give the rationale for the choice of cases and controls | Page 5-6 | |  |
|  |  | (b) For matched studies, give matching criteria and the number of controls per case |  | |  |
| Variables | 7 | Clearly define all outcomes, exposures, predictors, potential confounders, and effect modifiers. Give diagnostic criteria, if applicable | Page 6-7 | |  |
| Data sources/measurement | 8* | For each variable of interest, give sources of data and details of methods of assessment (measurement). Describe comparability of assessment methods if there is more than one group | Page 6-7 | | Same radiographic assessment protocol and PAI scoring system were applied to both denosumab-treated patients and healthy controls |
| Bias | 9 | Describe any efforts to address potential sources of bias | Page 5-7 | |  |
| Study size | 10 | Explain how the study size was arrived at | Page 6 | |  |
| Quantitative variables | 11 | Explain how quantitative variables were handled in the analyses. If applicable, describe which groupings were chosen and why | Page 6-7 | |  |
| Statistical methods | 12 | (a) Describe all statistical methods, including those used to control for confounding | Page 7 | |  |
|  |  | (b) Describe any methods used to examine subgroups and interactions | Page 7 | |  |
|  |  | (c) Explain how missing data were addressed | NA | |  |
|  |  | (d) If applicable, explain how matching of cases and controls was addressed | Page 5-6 | |  |
|  |  | (e) Describe any sensitivity analyses | NA | |  |
| **Results** |  |  |  | |  |
| Participants | 13* | (a) Report numbers of individuals at each stage of study—eg numbers potentially eligible, examined for eligibility, confirmed eligible, included in the study, completing follow-up, and analysed | Page 6 | | - Cases: 116 screened → 20 included  - Controls: 20 age- and sex-matched controls included |
|  |  | (b) Give reasons for non-participation at each stage | Page 6 | |  |
|  |  | (c) Consider use of a flow diagram | NA | |  |
| Descriptive data | 14* | (a) Give characteristics of study participants (eg demographic, clinical, social) and information on exposures and potential confounders | Page 7-8 | | Baseline demographic and clinical characteristics for cases and controls are presented in Table 1 (Page 20). |
|  |  | (b) Indicate number of participants with missing data for each variable of interest | NA | |  |
| Outcome data | 15* | Report numbers in each exposure category, or summary measures of exposure | Page 8-10 | | Outcome data were reported separately for the denosumab and control groups (Fig. 1A; Tab. 2 and 3). |
| Main results | 16 | (a) Give unadjusted estimates and, if applicable, confounder-adjusted estimates and their precision (eg, 95% confidence interval). Make clear which confounders were adjusted for and why they were included | Page 9-10 | |  |
|  |  | (b) Report category boundaries when continuous variables were categorized | Page 8 | |  |
|  |  | (c) If relevant, consider translating estimates of relative risk into absolute risk for a meaningful time period | NA | |  |
| Other analyses | 17 | Report other analyses done—eg analyses of subgroups and interactions, and sensitivity analyses | Page 9 | |  |
| **Discussion** |  |  |  | |  |
| Key results | 18 | Summarise key results with reference to study objectives | Page 10 | |  |
| Limitations | 19 | Discuss limitations of the study, taking into account sources of potential bias or imprecision. Discuss both direction and magnitude of any potential bias | Page 13-15 | |  |
| Interpretation | 20 | Give a cautious overall interpretation of results considering objectives, limitations, multiplicity of analyses, results from similar studies, and other relevant evidence | Page 10-15 | |  |
| Generalisability | 21 | Discuss the generalisability (external validity) of the study results | Page 14-15 | |  |
| **Other information** |  |  |  | |  |
| Funding | 22 | Give the source of funding and the role of the funders for the present study and, if applicable, for the original study on which the present article is based | Page 16 | |  |

*Give information separately for cases and controls.
